# The Importance of Frailty in Determining Survival After Intensive Care

**DOI:** 10.3390/jcm14051760

**Published:** 2025-03-05

**Authors:** Orsolya Szűcs, László G. Élő, Gábor Élő, Réka Varga, Réka Jung, Edit Benkovics, László Szabó, László Zubek

**Affiliations:** 1 Department of Surgery, Transplantation and Gastroenterology, Semmelweis University, Üllői Street 78, 1082 Budapest, Hungary; 2 Department of Anesthesiology and Intensive Therapy, Semmelweis University, Üllői Street 78, 1082 Budapest, Hungary; elo.laszlo@semmelweis.hu (L.G.É.); elo.gabor@semmelweis.hu (G.É.);; 3 Saint John’s Hospital, Kútvölgyi Outpatient Clinics, Kútvölgyi, Street 4, 1125 Budapest, Hungary

**Keywords:** frailty, intensive care unit, clinical frailty scale, long-term outcome, mortality

## Abstract

**Background:** Estimating survival and long-term quality of life after intensive care has been a crucial bioethical endeavour in recent decades to improve end-of-life decision-making. Scientific studies have also shown that patient frailty influences survival, but only a few long-term data are available. **Methods:** We conducted a prospective observational study at the Department of Anaesthesiology and Intensive Care of Semmelweis University, Hungary, to investigate the association between physical status on admission, the chance of survival, and the long-term quality of life of the patient. We recorded the pre-admission frailty score (Clinical Frailty Scale), APACHE II, and SAPS II scores on admission. The first follow-up was 3 months after discharge when the quality of life of the patient was assessed using the EQ5-D questionnaire. During the second follow-up one year later, we recorded the EQ5-D, Mini-Mental Test, and the Beck Depression Inventory scales. **Results:** Our study demonstrated that the ROC analysis of predicted overall mortality based on CFS score is similar in accuracy to that of predicted mortality by APACHE II and SAPS II point systems. The multivariate logistic regression calculations show that the best performing of the three independent variables is the SAPS II estimator (78.5%), but the estimators of both acute condition scoring systems (APACHE and SAPS) can be improved (79.5% vs. 84%) when taking into account the CFS value. The prevalence of mood and mental disorders among patients who survived one year was not different from that of the general population. **Conclusions:** The physiological scoring systems examined are all suitable for estimating the risk of overall mortality. The CFS shows similar efficacy and appears to be additive in value, with scales describing the severity of acute illness, which are indicative of the chronic condition of the patient.

## 1. Introduction

As medical science advances, physicians are faced with an ageing patient population due to increased life expectancy. Managing elderly patients has become a major challenge in recent decades. Beyond survival, the focus has shifted to achieving favourable long-term outcomes, ensuring quality of life, or sometimes limiting therapy in intensive care units (ICUs) for patients with a poor prognosis.

Alongside traditional hospital mortality prognostic scores, such as the Acute Physiology and Chronic Health Evaluation (APACHE) and the Simplified Acute Physiology Score (SAPS), the assessment of patient frailty at the onset of ICU treatment has become more common in recent years. Frailty is frequently observed in both hospitalised and ICU-admitted patients, and research indicates that it heightens the likelihood of adverse outcomes.

The scientific definition of frailty syndrome is as follows “A medical syndrome with multiple causes and contributors that is characterised by diminished strength, endurance, and reduced physiologic function that increases an individual’s vulnerability for developing increased dependency and/or death” [1]. The underlying idea behind Frailty Syndrome is that the cumulative adverse effects of ageing on various organs reduce the reserve capacity of the body. When this reserve falls below a certain threshold, the body has difficulty compensating for minor stressors—such as a new medication, minor trauma, or surgery—resulting in an increased risk of adverse reactions [2].

Risk factors such as age, gender, genetic problems, unhealthy lifestyle, inadequate nutrient and vitamin intake, and increased individual vulnerability lead to muscle weakness, chronic inflammatory conditions, and inadequate immune and endocrine response, which adversely affect the functioning of other organ systems, resulting in further loss of activity, fatigue, weight loss, and sarcopenia. The resulting vicious circle significantly impairs the ability of the patient to cope independently and to remain active [3,4].

The incidence of frailty increases exponentially with age, but it should be stressed that old age does not in itself equate to a frail state; frailty and old age are not synonymous, and pre-frail or frail states can also develop in the young. Young people typically recover more quickly after an adverse event, returning to the level of CFS before the onset of trauma/acute illness [5,6].

Co-morbidity is also not directly proportional to frailty; a well-adjusted diabetic patient who exercises regularly is not necessarily frail [7,8].

Several methods have been developed to detect and objectively quantify frailty, but we have briefly attempted to describe the two most commonly used scales. In the Cumulative Deficit Model, the Frailty Index (FI) is calculated by the number of deficits per individual/number of deficits recorded [3]. Several versions are known where at least 30 variables are recorded on patient’s lifestyle, co-morbidities, laboratory values, and physiological parameters. A meta-analysis by Kojima confirmed that FI can be used to predict mortality [9]; however, its recording is complicated and lengthy.

The Rockwood’s Clinical Frailty Scale (henceforth CFS) is a 9-point subjective scale that can be simplified from FI. It takes a few minutes to record, with simple questions to assess a patient’s activity, self-care, and fatigue. It correlates well with FI scores (r = 0.8) [10]. Pre-frailty is defined as a frailty prodrome for scores 3–4, and as a frailty syndrome (FS) for scores above 4. The modified 9-point CFS has been widely used for scoring patients in ICU studies, and the prognostic value of the CFS has been described as reliable in many publications, with the advantage of being easy to use. CFS has been studied in large numbers of cases to assess the morbidity of elderly patients. On ICU admission, both the precipitating frail conditions identified with FI and CFS have been associated with poor outcomes [11,12,13,14,15,16].

### Main Objectives of the Study

The primary objective of our study was to investigate the association between mortality and frailty in patients admitted to the ICU using the CFS and to compare the predictive value of CFS with scoring systems currently widely used to estimate mortality based on physiological parameters. We seek answers to the following question: Which factor significantly influences patient mortality, physical status on admission, or frailty status before admission? Our working group aims to develop a scoring system for patients referred to intensive care units that is able to predict, as accurately as possible, the chance of survival of these patients after being treated in this unit.

## 2. Materials and Methods

The Hungarian Frailty Study (HUF study) was a prospective observational study using validated questionnaires to examine factors influencing the survival of patients treated in Intensive Care Units (ICUs). The study was approved by the Ethics Committee of Semmelweis University (No. 217/2016, 24 October 2016).

All patients included in the study from 8 December 2016 to 2 February 2017 were admitted to two different intensive care units of the Department of Anaesthesiology and Intensive Care, Semmelweis University. Patients were followed up at 3 and 12 months. We aimed to include all patients admitted to the ICU during the study period, the only exclusion criterion being the lack of consent (Patients who did not wish to be included in the study either personally or by proxy were excluded).

### 2.1. Data Collection

All patients or their legal representative gave their informed consent to the study. Participation in the survey was voluntary, and consent could be withdrawn at any time, thereafter. Patients were subsequently identified anonymously by code number.

Data acquisition included patient demographics, date of admission, reason for admission, surgical (acute or elective) or internal medicine disease, and co-morbidities (pre-existing tumour, haematological disease, etc.).

We recorded the parameters for the calculation of the prognostic scoring systems (APACHE II and SAPS II) within 24 h of admission. The presence of frailty was assessed using the Clinical Frailty Scale (CFS), which reflects the patient’s condition 1 month before hospital admission, and, if necessary, the opinion of relatives was used to obtain a score as accurate as possible. This was recorded by the admitting physician in all cases. At discharge from the ICU, the number of days of care (ICU, ward) or date of death was also registered.

Two follow-up visits took place. First, patients were contacted 3 months after discharge, and the physician conducted a telephone interview to assess the quality of life of the 109 patients (EQ-5D). The one-year follow-up was conducted face-to-face, partly in the clinic and partly in the homes of the patients; however, several difficulties were encountered. A total of 17 patients had died, 57 of the 92 surviving patients were interviewed, 20 patients refused to fill in the questionnaires, the trauma of ICU treatment justifying their decision, 12 patients were unavailable or did not attend the interview despite the appointment, and 3 patients could not communicate with us properly. During the interview, we assessed patient mood, cognitive function, and quality of life (EQ-5D, SF-36, BDI and MMSE) among the survivors.

Clinical Frailty Scale (CFS): The CFS is a simple, quick-to-learn, bedside method of assessing a patient’s condition [10]. The scale ranges from 1 to 9, placing the patient between a very fit status and a state of complete dependence on others, inability to care for themselves, or terminal illness. It considers the chronic condition of the patient before hospitalisation rather than the sudden deterioration due to acute illness. In cases of discrepancy, we tried to rely on hetero-anamnesis.

Beck Depression Inventory (BDI): A 21-question questionnaire has been used worldwide to screen for pre-existing mood disorders since 1961 [17]. Several cut-off scores have been defined; in our study, we used the following: 0–10: normal; 11–16: mild mood disorder; 17–20: borderline clinical depression; 21–30: severe depression; >40: very severe depression.

Mini-Mental State Examination (MMSE): An 8-item scale for identifying cognitive function and dementia and assessing its severity, MMSE, was developed in 1975. The test is also used for screening cognitive impairment among ICU survivals [18,19]. A maximum of 30 points can be achieved, with no significant neuro-cognitive impairment above 24.

### 2.2. Statistical Analysis

All analyses were conducted using Python (version 3.10). Descriptive and inferential statistical techniques were employed to analyse patient data based on survival and other clinical parameters.

1. Descriptive Statistics
  ○ Continuous variables (e.g., age, ICU days, APACHE II, SAPS II, CFS scores) were summarised using means, standard deviations (SD), and medians with interquartile ranges (IQRs). Missing values were documented and excluded listwise for relevant analyses.
  ○ Categorical variables (e.g., gender, survival status) were reported as counts and proportions.
2. Group Comparisons
  ○ The Kruskal–Wallis test was used to compare continuous variables across multiple groups, such as CFS and survival categories, given the non-normal distribution of most variables.
3. Survival Analysis
  ○ Kaplan–Meier survival curves were generated to visualise survival probabilities over time, stratified by key clinical factors (e.g., CFS categories, gender).
  ○ Log-rank tests were performed to compare survival distributions between groups, including pairwise log-rank tests for multi-group comparisons.
  ○ Event definitions distinguished between patients who survived beyond one year (censored data) and those who experienced an event (death).
4. Chi-Squared Test
  ○ Associations between categorical variables, such as death categories and gender or CFS categories, were assessed using chi-squared tests of independence. Contingency tables and heatmaps were used to visualise these associations.
5. Visualisation
  ○ A variety of plots were created to visualise the data, including boxplots, density plots, and Kaplan–Meier survival curves.
6. Logistic Regression
  ○ Binary logistic regression was employed to assess the association between predictors (e.g., SAPS II, APACHE II, and CFS) and the binary outcome of survival status (alive or dead).

All statistical tests were two-tailed, and a *p*-value of less than 0.05 was considered statistically significant.

## 3. Results

**Figure 1:**
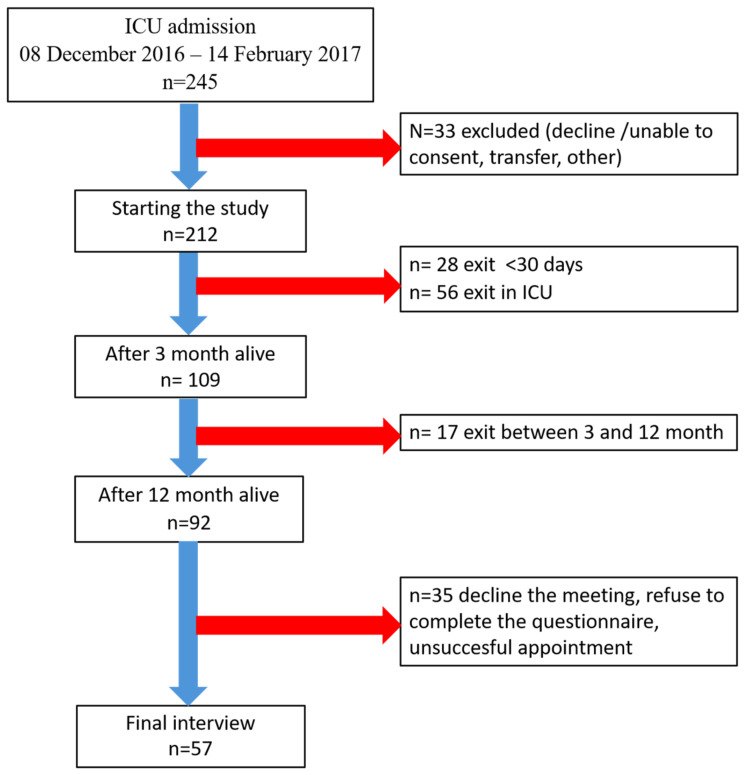
Flow chart of patient follow-up.

During the inclusion period, 245 patients were admitted to ICUs of the Department of Anaesthesiology and Intensive Therapy, Semmelweis University, 212 of whom (86.5%) were successfully enrolled following informed consent from patients or their close relatives. One year after ICU treatment, we were able to contact 92 patients, 57 of whom volunteered for a final follow-up. Engagement data are detailed in the flow chart below (Figure 1)

### 3.1. Demographic Data

In the total patient population studied, 59.4% were male, and 39.6% female. Among survivors, the proportion of men is slightly higher, at 64.1%, compared to 35.9% of women Table 1.

Of the patients admitted to the ICU, 65% were treated for an internal medicine indication and 35% for a surgical indication. In comparison, the proportions among survivors were 45.7% and 54.3%, so our data clearly show that those admitted for an internal medicine indication had a mathematically significantly higher (*p* < 0.001) mortality rate than those admitted for a surgical indication.

In the total patient population, the most common reason for admission was respiratory failure, for which 61 patients were admitted, of whom 1-year survival was 37.7%, and 34 patients who were treated for cancer, or its complications had a survival rate of 32.4%. In comparison, 32 patients were admitted for circulatory failure, 25% of whom had a 1-year survival rate. Fifteen patients were successfully resuscitated, but all patients died within six months of leaving the ICU. The data show that 43.4% of the 212 patients included were alive 1 year after their ICU treatment.

Based on our data, the SAPS II mean score was 39.72 (SD 19.76) and the APACHE II mean score was 16.18 (SD 9.0).

#### 3.1.1. Association with Survival

The Kruskal–Wallis test showed that both APACHE II and SAPS II scores were found to be significantly associated with patient mortality (*p* = 0.013 and *p* = 0.00013, respectively), with mortality estimated by the SAPS II score being more accurate based on our data.

Based on CFS scores, the mean score of admitted patients was 4.5 (SD 1.9), the mean score of those who died was 5.4 (SD 1.8), and the mean score of those who survived was 3.5 (SD 0.9).

**Figure 2:**
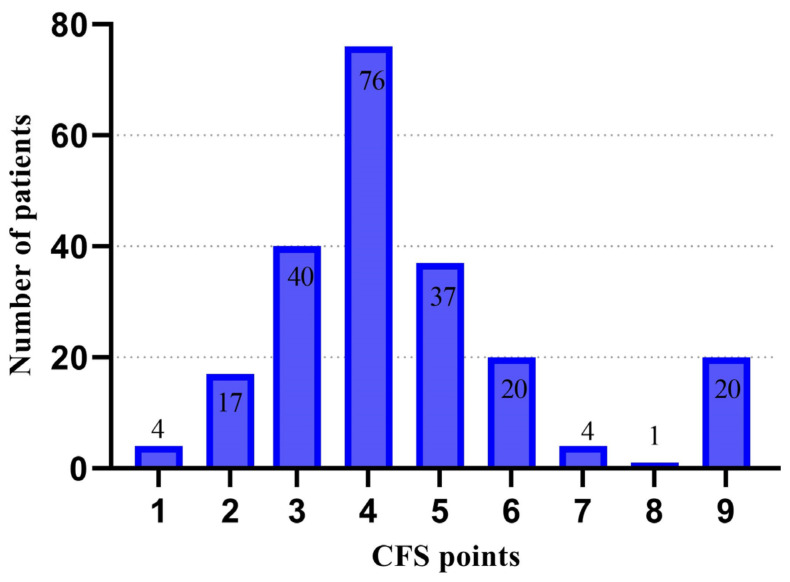
Distribution of CFS scores among patients admitted to the ICU.

Figure 2 below presents the distribution of admitted patients by CFS score.

**Figure 3:**
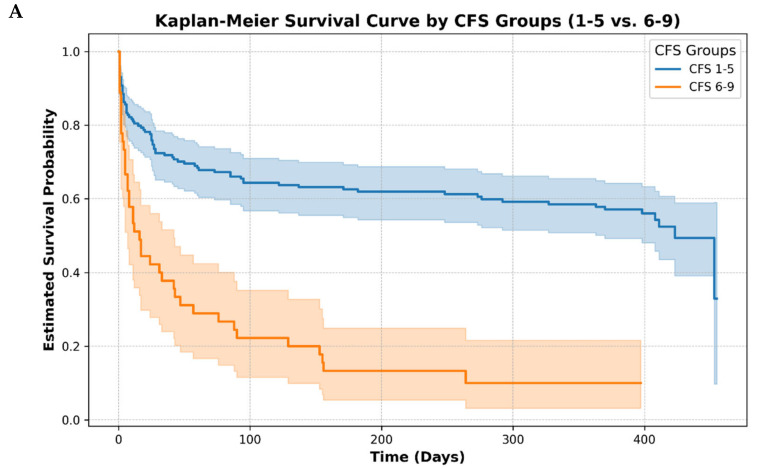

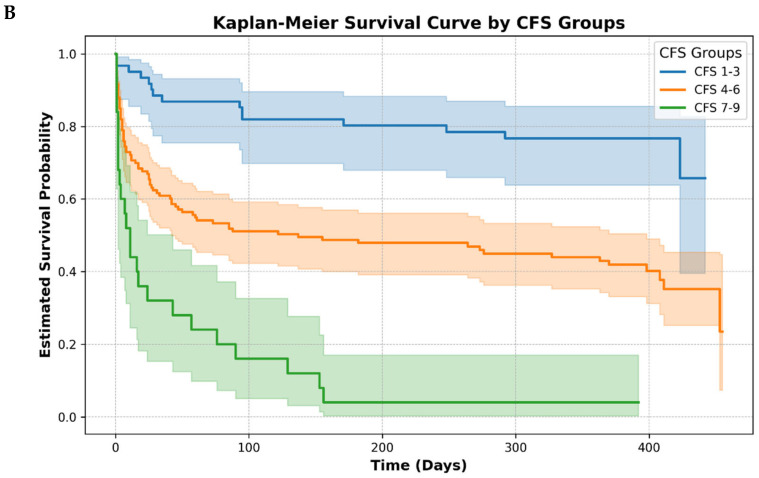
Kaplan–Meier survival curves for patients admitted, divided into groups of two and three based on CFS. The test comparing groups was based on log-rank test. The figure shows that the survival probability in groups CFS 1–5 (blue curve—(**A**)), CFS 1–3 (blue curve—(**B**)) was higher, compared to the other groups (orange line—(**A**), orange and green line—(**B**)).

To investigate the correlation between CFS scores and mortality of patients, we created groups of two (CFS: 1–4 and 5–9) or three (CFS: 1–3, 4–6, and 7–9) patients based on their CFS scores, and compared the mortality of each group. The log-rank test revealed a significant difference in survival probabilities between the different CFS groups (*p* < 0.0001), indicating that the CFS category was associated with variations in survival outcomes across the study population. In our study we found a significant correlation between CFS scores of patients and the risk of mortality in both the CFS subgroups of two and three, and a significant difference between the subgroups of two (CFS 1–4 and 5–9) and three (CFS: 1–3, 4–6, 7–9) in terms of total (early + late) mortality (*p* < 0. 01 and *p* < 0.00001). The Kaplan–Meier survival curve of the above grouping is presented in the following Figure 3.

#### 3.1.2. Correlation Between Scales

**Figure 4:**
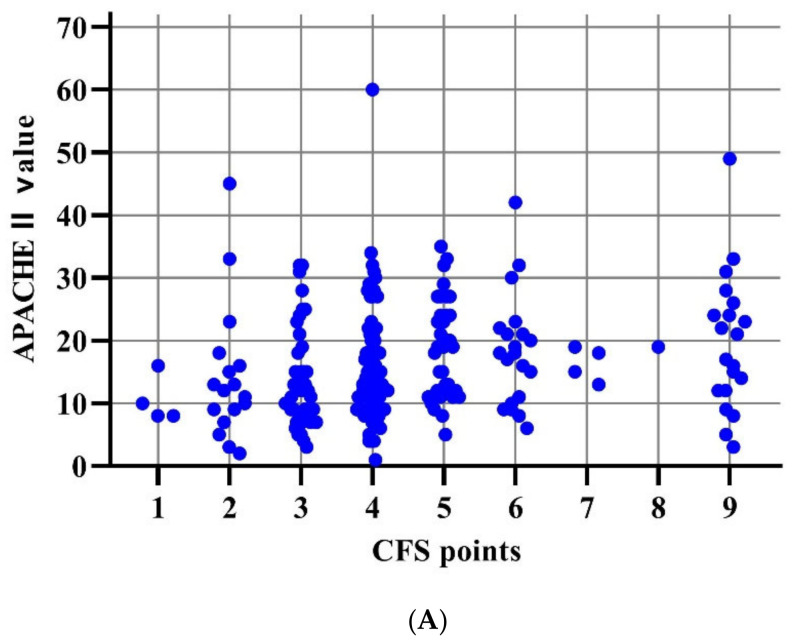

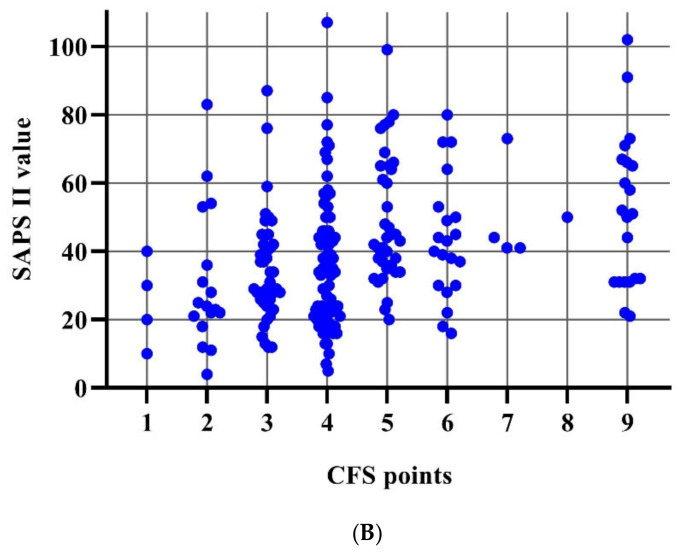
(**A**) Distributions of APACHE II by CFS. (**B**) Distributions of SAPS II by CFS.

The distribution of APACHE II and SAPS II scores across CFS scores varies widely as illustrated in the following Figure 4A,B. However, our data suggest that all three parameters (CFS, APACHE II, SAPS II) are useful predictors of mortality in practice; CFS scores vary independently of APACHE II values or SAPS II values.

The question arises as to whether there is any advantage to using CFS scores over the other two predictive scoring systems, in other words, whether or not there is any advantage to using CFS scores in predicting patient mortality. We compared the mortality risk in nine different CFS groups to clarify this.

**Figure 5:**
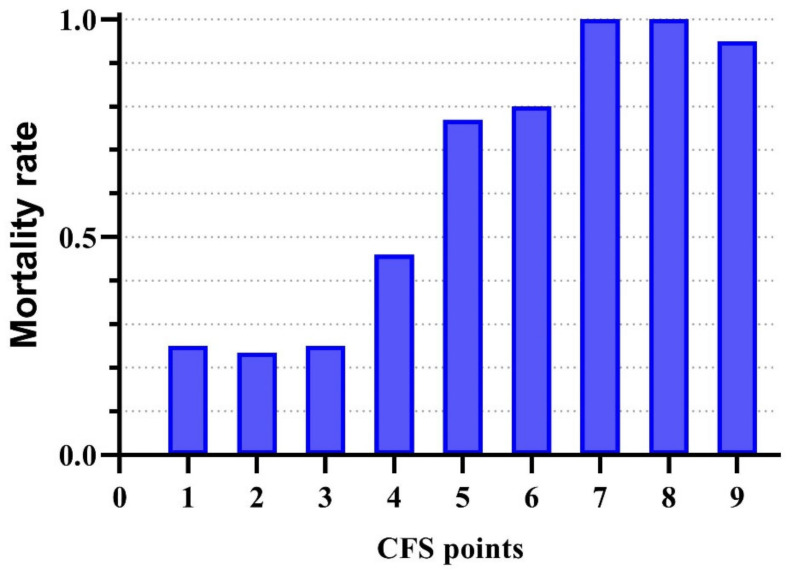
The mortality rate of all patients (*n* = 212) based on CFS points.

As can be seen in Figure 5, for low CFS scores (1–3), there is no relevant difference in mortality risk between the different CFS scores, with the risk of death varying around 25% across groups (23.5–25%), so that the occurrence of death is more likely to reflect the physiological impairment suffered. Our data showed that mortality was virtually inevitable for high CFS scores (7–9), so survival was much more strongly associated with CFS scores than APACHE II or SAPS II scores, indicating the degree of actual physiological impairment.

**Figure 6:**
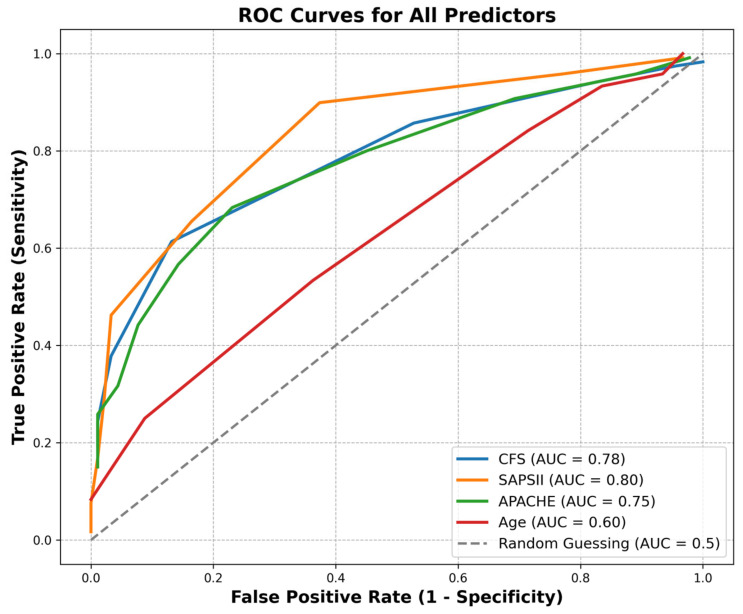
Prediction of mortality by CFS, APACHE II, SAPS II scores and age.

To evaluate the performance of each mortality prediction scoring system, we performed a Receiver Operating Characteristic (ROC) analysis based on our data. Figure 6. ROC analysis is widely used in research to survey the effectiveness of clinical scores. The ROC curve illustrates the discriminative ability of each predictor in distinguishing between patients with different clinical outcomes It helps identify optimal cut-off points by balancing true-positive rate (sensitivity) and false-positive rate (specificity). The 1.0 AUC value indicates perfect classification, 0.5 suggests no better achievement than random chance.

The area under the curve (AUC) values for the predictors were 0.78 using CFS, 0.75 using APACHE II, 0.80 using SAPS II, and 0.60 using age. SAPS II exhibited the highest discriminative ability (AUC = 0.80), followed closely by CFS (AUC = 0.78) and APACHE II (AUC = 0.75). Age had the lowest AUC (0.60), suggesting limited predictive value in this cohort. The dashed line (the 45-degree diagonal) represents random classification.

It presents the ROC curves for four clinical predictors: the Clinical Frailty Scale (CFS), the Simplified Acute Physiology Score II (SAPS II), the Acute Physiology and Chronic Health Evaluation II (APACHE II), and patient age. The results indicate that SAPS II and CFS may serve as robust predictors of patient outcomes, outperforming APACHE II and age in this analysis. Multivariate logistic regression was used to assess the sensitivity of the independent variables. The best predictive value when tested alone was the use of the SAPS (78.5%), followed by the predictive value of the APACHE score (71.7%), and then the CFS score (70.8%). However, when either the APACHE II or SAPS II score was examined in conjunction with the CFS score, the joint predictive accuracy increased, to 79.5% in the first case (APACHE + CFS), and 84% in the second case (SAPS + CFS). For details, see the Supplementary Materials.

### 3.2. Assessment of Patients’ Mood and Cognitive Function

In the group of patients surviving one year of intensive care, we obtained relevant data from 57 (62%) of the 92 (100%) survivors.

Among the late survivors, the mean MMSE scores were 27.9 points (SD: 1.5), compared to 27.2 (SD: 2.4) in a normal population based on the literature data, with the lowest recorded score being 25, so the cognitive function of all patients interviewed should be considered normal [20].

According to the Beck Depression Inventory questionnaire data, the mean score obtained was 2.52 (SD: 2.26), with a maximum measured score of 9, meaning that all responses were within the normal range.

We compared the different parameters constituting the APACHE and SAPS scoring systems between the surviving and dead patient groups. Our results showed no significant difference in any parameters (see Supplementary Materials).

## 4. Discussion

Several factors have been associated with frailty, with literature showing a higher proportion of women, people with multiple comorbidities, and smokers among frail patients. Prevalence is higher in patients over 50 years of age. Low education level, inactivity, depression, and antidepressant use also increase the prevalence of frailty [6,21].

Patients with a history of frailty before being admitted to the ICU already have reduced reserve capacity and, therefore, have a slower recovery and poorer quality of life after ICU treatment than their fit counterparts [22]

The literature suggests that up to a third of patients admitted to the ICU are in a frailty state; frailty is associated with higher in-hospital and long-term mortality. Some studies have found longer hospital stays, but not all studies have detected significant differences in the length of ICU care [5,8,23].

A prospective study of Kalaiselvan involving patients over 50 years of age found that patients with CFS ≥ 5 were older and had higher APACHE II scores on admission. He demonstrated longer ICU stays and higher 30-day mortality among frail patients [24]. Worse health status at six-month survival was reported among frail patients treated in the ICU for long periods [25].

Frailty is an independent risk factor for complications during ICU treatment and postoperative complications, and increases mortality in ICU patients, prolongs hospital stay and recovery, and increases the number of patients who are discharged from hospital to a place other than home. Several studies have confirmed that there may be higher rates of postoperative complications [26,27,28,29,30]. A meta-analysis by Hewitt demonstrated that morbidity was associated with the development of postoperative complications, longer hospital stays, and higher 30-day mortality in surgical patients [31].

In a Japanese study, CFS scores were recorded in patients over 65 years admitted from the Emergency Department to ICU, showing a correlation between 6-month mortality and quality of life. They confirmed that CFS was an independent prognostic factor for mortality at 6 months, and quality of life decreased inversely with the magnitude of the CFS score on admission [32].

Studies of patients undergoing elective vascular surgery suggest that morbidity increases the risk of death within 30 days of surgery and the risk of not being discharged home after hospitalisation. Preoperative recognition of frailty can also help plan goal-oriented surgical preparation and intervention [33].

A Hungarian study also focused on vascular surgery patients and supported the importance of preoperative screening for frailty. Using a comprehensive frailty index, Szabó et al. estimated the preoperative risk and long-term survival in cardiac and vascular surgery patients [34].

In geriatrics, several outcome studies have been published using the Frailty Syndrome. In a comprehensive geriatric survey in China and Canada, the status of elderly patients was assessed by a telephone interview using a modified CFS, and the test was found to be rapid and effective [35].

The VIP study (Very Old Intensive Care Patients) analysed data from approximately 5000 patients over 80 years of age. Their results also confirmed that a CFS score above 5 had a higher 30-day mortality, that a CFS recorded on ICU admission was a good predictor of premature mortality in elderly patients, and that it was recommended for its simple use in all elderly patients on admission and for consideration in decisions to limit treatment [7,36,37,38].

Shears has demonstrated in a prospective study that CFS is an easy-to-use screening tool in the ICU, with scores that can be reliably generated by the staff conducting or administering the research [39].

In a study published in 2011 that analysed a large population aged 15–102 years, Rockwood found that the prevalence of morbidity increased with age, but mortality was higher among less fit patients with higher FI scores at all ages and that the chance of full recovery from the disease decreased with advancing age [6].

In the literature review, the lower age limit for publications on frailty was generally between 50 and 65 years. In contrast, we included all patients admitted to the ICU who met the inclusion criteria without age restriction to screen young frail patients. Several previously published international studies have confirmed our hypothesis that young, critically ill cases would also have a worse outcome than if we only considered their chronological age. Frailty is a more accurate predictor of young patients than age; they have a lower reserve, and slower recovery than their non-frail peers of a similar age [13,27,40].

The above literature review supports the importance of assessing case fatalities; however, routine CFS recording has not yet been established in the ICU in Hungary.

Widely used, validated scoring systems (APACHE II, SOFA, SAPS II) that predict survival in ICU patients are based on acute physiological factors of patients that quantify disease severity and help caregivers predict the disease severity [40]. However, the predictive scoring system does not consider patient condition before hospitalisation or the onset of acute illness. Our data suggest that there is no significant correlation between APACHE II and SAPS II scoring systems and CFS scores, frailty being an independent predictor of mortality.

A prospective Swedish study examined the impact of premorbid frailty on 30-day mortality in patients treated in Intensive Care Units and compared CFS and SAPS 3 prognostic scales [13]. Based on their opinion “frailty needs to be recognised as a prognostic marker, and may be a valuable addition to established models for outcome prediction in intensive care”.

This study also supports our results that extend the previously used predictive scores to measure case fatality by adding CFS as appropriate. A meta-analysis by Bruno also confirmed that frailty (CFS ≥ 5) over 65 years is an independent factor for mortality. The cut-off value for young patients may differ from that for the elderly [41].

Based on our data, we believe that the low (1–3) and high (7–9) CFS scores are less differentially suitable for estimating mortality risk. Our data suggest that the mortality risk for CFS scores 1–3 is not significantly different, so their use does not provide additional information on outcomes. For high CFS scores, mortality is so high that patients do not survive hospitalisation regardless of the APACHE or SAPS scores. The curve plotting mortality as a function of CFS is, therefore, sigmoidal in shape, with the steepest part of the curve being between CFS scores 4 and 6. As our data shows, the Kaplan–Meier curve representing the overall mortality of all patients is closest to the curve for patients with a CFS = 5. From our point of view, we believe that, in comparison, a CFS score of 4 could represent a 15–20% reduction in the risk of death, and a CFS score of 6 could increase mortality risk by 15–20%. Of course, a larger sample of patients is needed to determine a more precise correlation, with a larger number of patients from each CFS group compared to our study. We included patients in our observational study indiscriminately, unlike other studies, we also included young patients.

The ROC analysis is an illustrative method for testing and comparing the effectiveness of diagnostic tests, providing information on test specificity and sensitivity. Our study compared the predictive value of age, APACHE II, SAPS II, and CFS scores. The study results indicate that the SAPS II score is the most accurate predictor of mortality (AUC: 0.80), followed by the CFS (AUC: 0.78) and then the APACHE II score (AUC: 0.75). The higher AUC values for SAPS II and CFS suggest that these scores have better sensitivity and specificity in distinguishing between survival outcomes. We found that the correlation of mortality with age is very weak (AUC: 0.60) and, therefore, cannot be used in clinical practice to estimate the chance of mortality. Our calculation, based on the multivariate logistic regression, shows that the highest accuracy is obtained by using two independent variables (CFS and SAPS II score) together, with an accuracy of 84%, which is already acceptable.

Kaplan–Meier curves show the difference in the distribution of the number of survivors after dividing the patient population into groups of two and three (Figure 3).

In the early stages, FS and pFS may be reversible, as both physical and mental well-being can be improved by optimising exercises or medication or by treating an addiction. For prevention reasons, routine CFS score recording at any doctor–patient encounter over 65 years of age would be justified, so GPs and anaesthesia outpatients would have a crucial role to play. In patients awaiting elective major surgery, a comprehensive assessment of morbidity is recommended, regardless of age. Mitigating the risk will also improve patient safety.

### Cognitive Decline

Post-intensive care syndrome (PICS) is defined as a new or worsening cognitive, psychiatric, or physical function after a critical illness or discharge from the ICU. ICU survivors may have physical, emotional, and mental symptoms. Moreover, the survivors often report fatigue and pain [42].

The elderly population is affected not only physically but also mentally. Studies have demonstrated a bilateral association between depression, dementia, and frailty, but it is unclear whether this is an independent risk factor or part of FS. Activity decline and risk of FS are more common in depressed older people. In 2013, a consensus group specifically addressed the issue of cognitive frailty [43,44].

In our study of cognitive frailty, the BDI and MMSE tests of late survivors showed standard scores, meaning that we did not observe a higher incidence of depression or cognitive decline in ICU survival population than in the general population. However, we could only examine 62% of the long survival group. The rationale for late-interview refusers raises the possibility of PICS, but this has not been proven yet. Future studies would benefit from investigating the additive impact of premorbid mood disorder or cognitive decline on mortality rate. This is a challenging problem to solve, as there are currently no BDI or MMSE data on patients before ICU treatment to which the post-ICU status can be compared, so that these parameters can be examined as independent predictors of mortality. The design and implementation of such a study would require enormous resources, involving tens of thousands of patients, a small percentage of whom will be admitted to intensive care during the study period, or a very long study period of several decades with fewer patients.

## 5. Conclusions

Frailty is common among patients admitted to the Intensive Care Unit (ICU). It impairs quality of life and increases the risk of adverse, even fatal outcomes, and may therefore play a role in end-of-life decision-making. The scoring systems used to estimate mortality in the ICU (APACHE II, SAPS II) focus on acute deterioration and, therefore, do not measure the pre-hospitalisation status.

Our investigation has found, consistent with previous evidence, that CFS is an independent predictor of late mortality, with accuracy falling between SAPS II and APACHE II scoring systems in predicting mortality. It is primarily most sensitive at intermediate CFS scores (4–6) and the most accurate predictor of mortality at high CFS scores (7–9), independent of an acutely developed critical state. If the risk is recognised early, an individualised treatment plan involving the family and the general practitioner can increase the chances of quality survival. During intensive care unit treatment, frail patients should be closely monitored, as early mobilisation, weaning, and adequate feeding can help these patients recover more quickly. CFS is a suitable parameter because it is easy to use and can be assessed even when the patient can no longer make a statement based on hetero-anamnesis alone. Although CFS alone could be a reliable test for estimate mortality, we believe that combining it with commonly used prognostic tests has great advantages. Assessing state of consciousness or mood can also be important in estimating survival, but it is much more challenging to measure. Further analysis is planned to demonstrate the relationship between frailty, mental health, and ICU survival and to improve the understanding of physical and mental frailty.

### Strengths and Weaknesses

The strength of our study is that data were collected prospectively, using a mixed (internal medicine and surgery) ICU patient population. The three-month follow-up was successful; all survivors were surveyed using a questionnaire. During the study period, 86.5% of patients admitted to the intensive care unit were included without selection.

Our study has several limitations. First, it was a single-centre study; only patients admitted to two intensive care units of Semmelweis University, the Department of Anaesthesiology and Intensive Therapy were investigated, so the results are not representative.

Another limitation of the study is that the quality of life of patients before admission was often determined retrospectively, along with their hetero-anamnesis. The number of patients in the different CFS groups was not equal, with rare patients with CFS = 1 and CFS 7–8 scores. The large number of patients with a CFS score of 9 is also due to the difficulty in practice in distinguishing between CFS 8 and 9. The CFS level was assessed by the treating physician of the patient, rather than an independent researcher. Some unconscious bias may occur as a result of the observational study. Further validation in larger cohorts is warranted to confirm these findings and refine risk stratification models.

Finally, it was not possible to interview all survivors at the one-year follow-up, so data on 38% of the patients were lost. The high proportion of patients refusing late interviews may indicate post-traumatic reactions in these patients.

## Figures and Tables

**Table 1:** Demographics, reasons for admission, scores for severity of illness, outcomes.

	Admitted Patients	Death Within 1 Year	Alive at 1 Year
Admitted patients	212	120	92
Age (years) mean	67.1	70.5	63.4
Age (years) median	68	70	66
Sex: male (n)	126	67	59
female (n)	86	53	33
Reason for admission			
Oncology disease	34	23	11
Cardiovascular disease	32	24	8
Respiratory failure	61	38	23
Treated with an internal medicine diagnosis	138	96	42
Treated with a surgical diagnosis	74	24	50
St.p. CPR	15	15	0
ICU LOS mean (day) *	6	6,9	5
ICU LOS median (day)	4	4	4
Severity of illness			
CFS mean (SD)	4.5 (1.9)	5.4 (1.7)	3.5 (0.9)
CFS median	4	5	4
APACHE-II mean (SD) **	16.18 (9.0)	20.52 (7.39)	11.51 (4.17)
APACHE-II on ICU admission median	14	19	11
SAPS-II mean (SD) ^#^	39.72 (19.76)	50.95 (15.64)	28.42 (9.53)
SAPS-II on ICU admission median	36.5	46	26.5

Abbreviations: * ICU LOS: length of stay in the Intensive Care Unit, ^#^ SAPS II: Simplified Acute Physiology Score II, ** APACHE II: Acute Physiology and Chronic Health Evaluation II.

## Data Availability

Data are contained within the article.

## Supplementary Materials

The following supporting information can be downloaded at: https://www.mdpi.com/article/10.3390/jcm14051760/s1, Figure S1: Length of stay in the Intensive Care Units depending on clinical predictor scores. Table S1: Physiological parameters (APACHE and SAPS) of living and dead patients. Table S2: Multivariate logistic regression.

## Abbreviations

CSF	Clinical Frailty Scale
ICU	Intensive Care Unit
PICS	Post-Intensive Care Syndrome
VAS	Visual Analogue Scale
pFS	Pre-Frailty Syndrome
SAPS II	Simplified Acute Physiology Score II
APACHE II	Acute Physiology and Chronic Health Evaluation II
ROC	Receiver Operating Characteristic
LOS	Length of Stay
