# Supplementary material for: The Importance of Frailty in Determining Survival After Intensive Care
Source: J Clin Med. 2025 Mar 5;14(5):1760. doi: 10.3390/jcm14051760 (PMC11900552; doi:10.3390/jcm14051760)
Supplement: Supplementary file 1 [file jcm-14-01760-s001.zip › jcm-3430661-supplementary.pdf]

Supplementary Materials:

Table S1. Physiological parameters (APACHE and SAPS) of living and dead patients

Death	Variable	Mean	SD	Min	Q1	Median	Q3	Max	Missing
dead	Age	70.53	13.3	31.0	63.0	70.0	79.75	95.0	0
dead	CFS	5.27	1.99	1.0	4.0	5.0	6.0	9.0	0
dead	ICU days	6.8	7.39	1.0	2.0	4.0	8.0	35.0	0
dead	APACHE II	20.19	9.52	3.0	13.0	19.0	27.0	60.0	0
dead	SAPS II	49.72	19.63	10.0	35.0	45.0	64.0	107.0	0
dead	Body Temperature	36.77	1.51	29.9	36.0	37.0	37.7	40.0	1
dead	MAP*	69.54	20.22	35.0	58.0	65.0	78.0	129.0	1
dead	Arterial pH	7.32	0.15	6.85	7.27	7.34	7.41	7.65	1
dead	HR**	94.84	33.34	40.0	65.0	91.0	121.0	170.0	1
dead	Resp	19.27	6.99	0.35	15.0	18.0	24.0	45.0	1
dead	Natrium	138.88	5.99	118.0	136.0	139.0	143.0	156.0	2
dead	Potassium	4.0	0.88	2.5	3.4	3.87	4.4	6.44	3
dead	Creatinin	149.46	134.44	7.7	71.0	109.0	172.0	857.0	1
dead	Hematocrit	0.35	0.08	0.17	0.31	0.34	0.4	0.57	2
dead	WBC	12.05	8.17	0.01	7.13	10.57	15.6	43.0	1
dead	Systolic BP[#]	114.71	43.14	30.0	83.0	101.0	150.0	229.0	1
dead	PaO2	104.69	51.04	37.1	70.0	92.1	121.0	383.0	1
dead	PCO2	40.65	17.46	3.2	29.4	36.9	46.1	118.0	1
dead	Diuresis	1584.53	1059.08	0.0	750.0	1475.0	2225.0	4500.0	2
dead	BUN	12.85	8.71	0.7	6.57	10.0	17.97	47.1	2
dead	HCO3-	21.2	5.93	4.7	17.9	20.8	24.5	37.4	1
dead	Bilirubin	19.01	17.47	2.3	9.3	13.3	21.9	112.0	12
alive	Age	62.63	15.11	19.0	56.0	66.0	72.0	91.0	0
alive	CFS	3.57	1.19	1.0	3.0	4.0	4.0	9.0	0
alive	ICU days	5.26	4.96	1.0	2.0	4.0	6.0	25.0	0
alive	APACHE II	11.43	5.35	1.0	8.0	11.0	14.0	31.0	0
alive	SAPS II	28.03	12.03	4.0	20.0	25.0	37.0	71.0	0
alive	Body Temperature	36.47	1.28	33.8	35.6	36.5	37.4	39.1	0
alive	MAP	80.97	19.71	40.0	67.0	78.0	95.0	133.0	0
alive	Arterial pH	7.36	0.08	7.09	7.31	7.35	7.4	7.64	0
alive	HR	82.87	25.64	36.0	60.0	77.0	105.0	150.0	0
alive	Resp	17.31	7.03	10.0	14.0	15.0	18.0	46.0	0
alive	Natrium	138.26	6.25	110.0	136.0	140.0	141.0	149.9	0
alive	Potassium	3.98	0.81	2.2	3.49	3.83	4.43	7.0	1
alive	Creatinin	98.92	87.39	4.6	59.0	73.5	110.25	674.0	3
alive	Hematocrit	0.36	0.06	0.21	0.32	0.36	0.39	0.52	2
alive	WBC	12.9	12.83	3.25	8.2	10.08	14.06	123.0	1
alive	Systolic BP	131.91	38.24	70.0	97.0	130.0	160.0	220.0	0
alive	PaO2	108.14	44.26	36.7	79.0	96.6	127.0	318.0	0
alive	PCO2	39.36	13.34	14.6	33.4	38.6	43.2	121.0	0
alive	Diuresis	2151.49	899.57	0.0	1500.0	2200.0	2600.0	5500.0	0
alive	BUN	8.37	10.85	1.4	4.12	5.8	8.78	96.0	3
alive	HCO3-	21.82	4.5	9.6	19.5	21.3	23.9	34.1	4
alive	Bilirubin	13.98	9.54	1.5	8.0	12.0	17.0	66.0	12

*MAP: Mean Arterial Pressure **HR: Heart Rate [#]BP: Blood Pressure

Figure S1. Length of stay in the Intensive Care Units depending on clinical predictor scores.

A

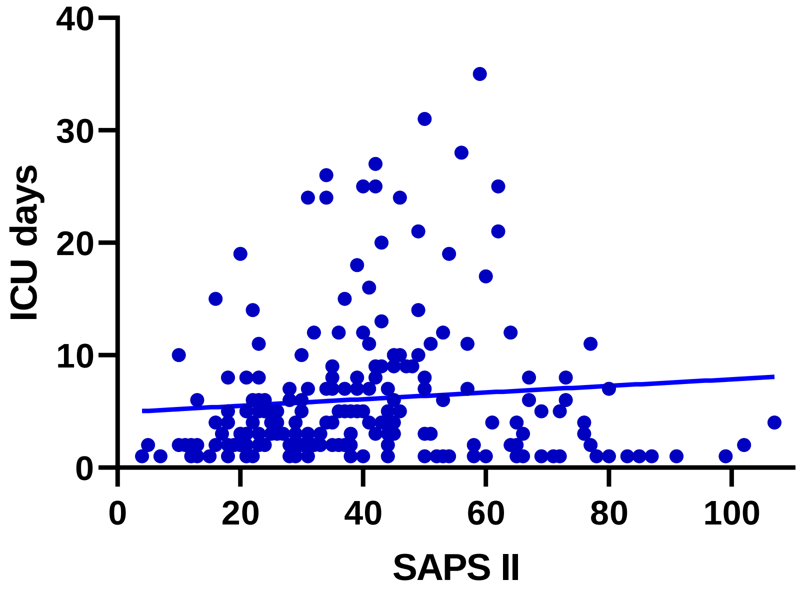

B

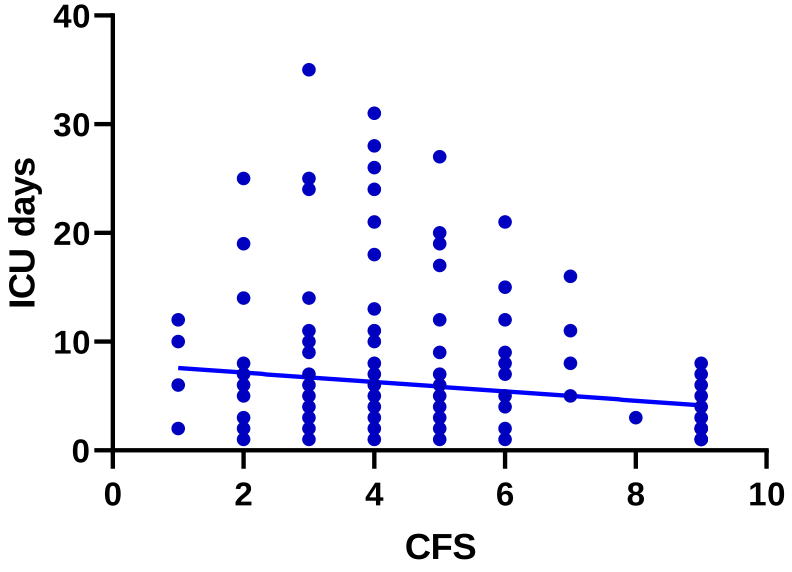

C

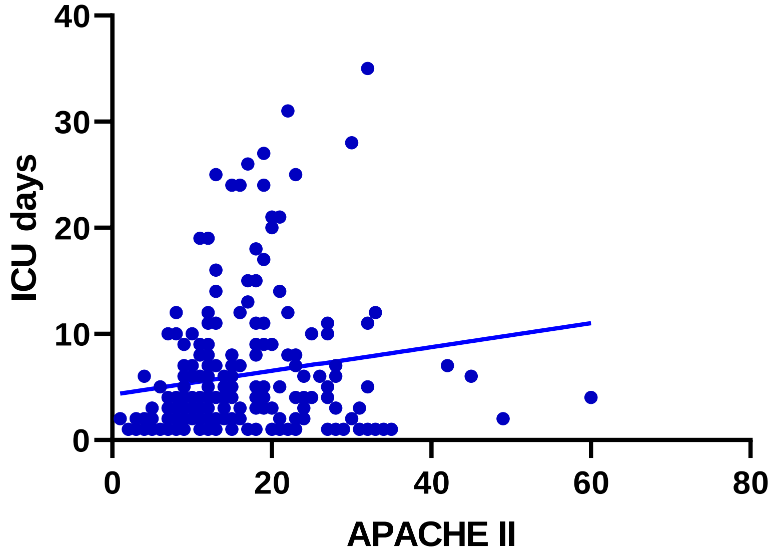

Figure S1 A: Length of stay in the Intensive Care Units (days) depending on SAPS II, B: CFS, C: APACHE II score. No significant correlation was found.

Table S2. Multivariate logistic regression calculation:

	-2 Log likelihood	Cox & Snell R Square	Nagelkerke R Square	Percentage Overall correct classification[a]
**SAPS II**	206,385	0,355	0,474	78,5
**CFS**	241,931	0,241	0,322	70,8
**APACHE**	237,503	0,256	0,342	71,7
**CFS+SAPS II**	**144,124**	**0,514**	**0,687**	**84**
**CFS+APACHE**	188,354	0,406	0,542	79,5

Model summary of logistic regression test and the percentage of overall correct survival status classifications using different scoring systems or their combinations. (a) the cut value is 0,500
